# Clinical factors of post-chemoradiotherapy as valuable indicators for pathological complete response in locally advanced rectal cancer

**DOI:** 10.6061/clinics/2016(08)07

**Published:** 2016-08

**Authors:** Jianhong Peng, Junzhong Lin, Miaozhen Qiu, Xiaojun Wu, Zhenhai Lu, Gong Chen, Liren Li, Peirong Ding, Yuanhong Gao, Zhifan Zeng, Huizhong Zhang, Desen Wan, Zhizhong Pan

**Affiliations:** I Sun Yat-sen University Cancer Center, State Key Laboratory of Oncology in South China, Collaborative Innovation Center of Cancer Medicine, Department of Colorectal Surgery; II Sun Yat-sen University Cancer Center, State Key Laboratory of Oncology in South China, Collaborative Innovation Center of Cancer Medicine, Department of Medical Oncology; III Sun Yat-sen University Cancer Center, State Key Laboratory of Oncology in South China, Collaborative Innovation Center of Cancer Medicine, Department of Radiation Onology; IV Sun Yat-sen University Cancer Center, State Key Laboratory of Oncology in South China, Collaborative Innovation Center of Cancer Medicine, Department of Pathology, Guangzhou, P.R. China

**Keywords:** Rectal Cancer, Pathological Complete Response, Preoperative Chemoradiotherapy, Prediction

## Abstract

**OBJECTIVES:**

Pathological complete response has shown a better prognosis for patients with locally advanced rectal cancer after preoperative chemoradiotherapy. However, correlations between post-chemoradiotherapy clinical factors and pathologic complete response are not well confirmed. The aim of the current study was to identify post-chemoradiotherapy clinical factors that could serve as indicators of pathologic complete response in locally advanced rectal cancer.

**METHODS:**

This study retrospectively analyzed 544 consecutive patients with locally advanced rectal cancer treated at Sun Yat-sen University Cancer Center from December 2003 to June 2014. All patients received preoperative chemoradiotherapy followed by surgery. Univariate and multivariate regression analyses were performed to identify post-chemoradiotherapy clinical factors that are significant indicators of pathologic complete response.

**RESULTS:**

In this study, 126 of 544 patients (23.2%) achieved pathological complete response. In multivariate analyses, increased pathological complete response rate was significantly associated with the following factors: post-chemoradiotherapy clinical T stage 0-2 (odds ratio=2.098, 95% confidence interval=1.023-4.304, *p*=0.043), post-chemoradiotherapy clinical N stage 0 (odds ratio=2.011, 95% confidence interval=1.264-3.201, *p*=0.003), interval from completion of preoperative chemoradiotherapy to surgery of >7 weeks (odds ratio=1.795, 95% confidence interval=1.151-2.801, *p*=0.010) and post-chemoradiotherapy carcinoembryonic antigen ≤2 ng/ml (odds ratio=1.579, 95% confidence interval=1.026-2.432, *p*=0.038).

**CONCLUSIONS:**

Post-chemoradiotherapy clinical T stage 0-2, post-chemoradiotherapy clinical N stage 0, interval from completion of chemoradiotherapy to surgery of >7 weeks and post-chemoradiotherapy carcinoembryonic antigen ≤2 ng/ml were independent clinical indicators for pathological complete response. These findings demonstrate that post-chemoradiotherapy clinical factors could be valuable for post-operative assessment of pathological complete response.

## INTRODUCTION

Currently, preoperative chemoradiotherapy (CRT) followed by total mesorectal excision (TME) is the standard treatment for patients with locally advanced rectal cancer (LARC) to improve local disease control and sphincter preservation rate 1,2. Following preoperative chemotherapy, variable responses have been observed among individuals, with a range of 12% to 40% of cases achieving pathological complete response (pCR) 3-5. Patients achieving pCR after CRT have a lower incidence of recurrence and more favorable long-term survival than those without pCR 6,7. Additionally, due to the associated relatively long-term survival, tumor complete response allows the adoption of a “wait-and-see” policy for patients undergoing neoadjuvant CRT instead of necessitating immediate surgery 8,9. Therefore, it is of considerable clinical importance to identify significant factors that can successfully predict pCR after preoperative CRT. Although many techniques have been applied towards this goal, correlations between post-CRT clinical factors and pCR are seldom reported and remain poorly defined 10. To identify significant post-CRT clinical factors associated with pCR, we conducted the current study using a large cohort of patients undergoing CRT and surgery at a single center.

## PATIENTS AND METHODS

### Patient selection

This study included a total of 577 consecutive patients with rectal cancer who underwent preoperative CRT followed by surgery from December 2003 through June 2014 at Sun Yat-sen University Cancer Center, China. The following inclusion criteria were applied: (1) histologically confirmed rectal adenocarcinoma; (2) cT3-4 or N+ disease before CRT; (3) no other anti-tumor therapy was received before CRT; and (4) no distant metastatic disease. Thirty-one patients were excluded from this study due to distant metastatic disease before CRT and an additional two patients who were confirmed to have adenosquamous carcinoma were also eliminated. This left a total of 544 individuals who were included for analysis. The detailed clinical data of the eligible patients were reviewed using our electronic medical record system. Prior to CRT and surgery, informed consent was obtained from each patient. The study was undertaken in accordance with the ethical standards of the World Medical Association Declaration of Helsinki and study approval was obtained from independent ethics committees at Sun Yat-Sen University Cancer Center.

### Treatment

All of the subjects underwent preoperative CRT. Radiation therapy (RT) was administered via three-dimensional conformal radiation therapy (3D-CRT) or intensity-modulated radiation therapy (IMRT). The patients were scheduled to receive a total irradiation dose of 46.0-50.4 Gy to the pelvic area, delivered in 1.8-2.0 Gy fractions daily on five consecutive days per week for 5-6 weeks. If a patient could not tolerate the full dose or suffered from severe toxic effects, the RT was stopped. Patients who were reluctant to undergo surgery were delivered a radical dose of RT.

One of the following chemotherapeutic regimens was delivered concurrently with the RT: (1) XELOX regimen consisting of 130 mg/m^2^ oxaliplatin administered intravenously on day 1 and 1,000 mg/m^2^ capecitabine administered orally twice daily on days 1-14 for a 3-week cycle; (2) FOLFOX regimen consisting of 85 mg/m^2^ oxaliplatin and 400 mg/m^2^ leucovorin administered intravenously over 2 hours on day 1, 400 mg/m^2^ 5-FU administered intravenously on day 1 and 1200 mg/m^2^ 5-FU administered intravenously for 2 days for a 2-week cycle; or (3) 825 mg/m^2^ capecitabine administered orally twice daily during RT without weekend breaks.

All subjects were scheduled for radical resection 6–8 weeks after the completion of preoperative CRT. If bowel obstruction or perforation occurred after CRT, emergent surgery was performed immediately. Surgery was delayed in the event of grade 3 or 4 toxicity. The surgical procedure used depended on tumor location and invasive extent; TME was performed whenever possible.

### Evaluation

Post-CRT clinical evaluation was performed at the 6^th^ week after completion of CRT and included a combination of physical examination, post-CRT carcinoembryonic antigen (CEA) test, colonoscopy, transrectal ultrasonography (EUS), chest computed tomography (CT) and abdominal and pelvic nuclear magnetic resonance (MRI). Disease stage was classified according to the 2010 American Joint Committee on Cancer/International Union Against Cancer (AJCC/UICC) staging system. According to EUS images, post-CRT clinical (yc) T stage 0 was defined as no tumor signs in the bowel wall; ycT stage 1 was defined as a tumor signal confined to the submucosa; and ycT stage 2 was defined as a malignancy that had invaded the muscularis propria. Using MR images, ycN stage 0 was defined as the absence of metastatic lymph nodes; ycN stage 1 and ycN stage 2 were respectively defined as the presence of 1-3 and >3 metastatic lymph nodes, which appeared irregular in shape, showed enhancement and possessed a diameter >5 mm; ycT stage 3 was defined as a malignancy that broke through the muscularis propria; and ycT stage 4 was defined as a tumor that had invaded adjacent organs. All radiological reports were confirmed by two independent radiologists. Postoperative final tumor pathological staging was also evaluated by two independent pathologists. pCR was defined as the absence of viable tumor cells, with only fibrotic masses or acellular mucin pools present in proximity to the primary tumor and lymph nodes 11. Patients confirmed as having achieved pCR were classified as the pCR cohort, while the remaining patients formed the non-pCR cohort.

### Statistical analysis

Data were analyzed using SPSS version 17.0 (Chicago, IL, USA). Continuous variables were shown as the median (range) or the mean (standard deviation) and categorical variables were presented as percentages. All continuous and polytomous variables were dichotomized by a cut-point prior to univariate and multivariate analyses. Univariate comparisons of factors were performed using the chi square test or Fisher’s exact test for categorical variables and an unpaired t-test for continuous variables. Factors with statistical significance in univariate analyses were included in multivariate analyses. Multivariate analyses were performed using logistic regression to identify significant post-CRT clinical indicators and therapeutic factors for pCR. All tests were two-tailed and *p*<0.05 was considered statistically significant.

## RESULT

### Clinical demographics

A total of 544 patents, 361 men (66.4%) and 183 women (33.6%) were enrolled. The median patient age was 55 years (range 12-81 years). Before CRT, 170 patients (31.1%) presented clinical stage II disease and 374 (68.7%) presented clinical stage III disease. The clinical demographics and treatment results are presented in Table 1. The median radiation dose was 50 Gy (range 30–70 Gy). Regarding the concurrent chemotherapy, 473 (87.0%) patients received the XELOX regimen, while 49 (9.0%) patients received the FOLFOX regimen, and 22 (4.0%) were given oral capecitabine alone. After CRT, the median distance of the inferior tumor margin from the anal verge (DAV) was 5 cm (range 0–15 cm) and the median interval from completion of CRT to surgery was 51 days (range 7–78 days). All patients underwent surgery: 318 (58.5%) underwent anterior resection, 196 (36.0%) underwent abdominal perineal resection, 14 (2.6%) underwent the Hartmann procedure and the remaining 16 (2.9%) patients underwent unsuccessful tumor resection and subsequently received palliative colostomy. After the comprehensive treatment, 126 patients (23.2%) obtained pCR. The general clinical parameters, including age, gender, pre- and post-CRT DAV, pre- and post-CRT body mass index (BMI), pre-CRT clinical tumor-node-metastasis (TNM) stage, surgical procedure, radiation pattern and chemotherapy regimens, were comparable between the pCR cohort and the non-pCR cohort. Radiation dose (*p*=0.041), yc T stage (*p*=0.002), yc N stage (*p*=0.002), ycTNM stage (*p*=0.001), tumor differentiation (*p*=0.001), median interval from completion of CRT to surgery (*p*=0.007) and post-CRT CEA level (*p*=0.011) were significantly different between the two groups.

### Post-CRT clinical indicators for pCR

The cut-off values for post-CRT CEA, DAV and interval from the completion of CRT to surgery were set according to the median values. In the univariate analyses, the potential post-CRT clinical indicators included ycT stage, ycN stage, post-CRT DAV, interval from completion of CRT to surgery, radiation dose and post-CRT CEA level (Table 2). The multivariate logistic regression analyses indicated that ycT stage 0-2 [odds ratio (OR)=2.098, 95% confidence interval (CI)=1.023-4.304, *p*=0.043], ycN stage 0 (OR=2.011, 95% CI=1.264-3.201, *p*=0.003), interval from completion of CRT to surgery of >7 weeks (OR=1.795, 95%CI=1.151-2.801, *p*=0.010) and post-CRT CEA≤2 ng/ml (OR=1.579, 95%CI=1.026-2.432, *p*=0.038) were independent factors for increased pCR (Table 3).

## DISCUSSION

Unlike the majority of previous studies, which have focused on pre-CRT factors that can predict pCR 12,13, the present study evaluated the value of certain post-CRT parameters and treatment variables for predicting pCR. Using univariate and multivariate analyses, we showed that ycT stage 0-2, yc N stage 0, interval from completion of CRT to surgery of >7 weeks and post-CRT CEA ≤2 ng/ml could be applied to assess pCR post-operatively.

In our study, a favorable pCR rate of 23.2% was achieved, which was comparable to previous reports in which CRT was delivered with fluoropyrimidine-based chemotherapy combined with oxaliplatin 4,14,15. To improve pCR rates, several randomized trials have been conducted to integrate oxaliplatin into the currently widely used fluoropyrimidine-based preoperative chemotherapy regimen. The majority of these trials have achieved a favorable pCR rate, ranging from 28% to 40% 5,16. However, another phase III study showed that the addition of oxaliplatin into CRT did not increase pCR: the pCR rate in this study was 13.9% in the capecitabine group compared to 19.2% in the capecitabine plus oxaliplatin group (*p*=0.09) 17. To the best of our knowledge, drug dose, patient tolerance during treatment and administration schedule all influence the achievement of pCR. A better understanding of chemotherapy schedule as well as the features of specific cohorts of patients might contribute to increase pCR after CRT.

CEA is not only a prognostic factor but also a predictor for response to preoperative CRT in LARC 18. Pretreatment CEA level has been demonstrated as a useful marker for pCR following preoperative CRT 13,19. However, few studies have evaluated the value of post-CRT CEA level in assessing pCR following preoperative CRT. Yang et al. evaluated the association of CEA level and pCR rate in 138 patients treated with preoperative CRT and found that a post-CRT CEA level <2.61 ng/ml could indicate pCR postoperatively (OR=0.605, 95%CI=0.412-0.890, *p*=0.011)^20^. Kleiman et al. showed that post-CRT CEA levels were significantly lower in patients with pCR (1.7 *vs*. 2.4 μg/L, *p*<0.01), indicating the normalization of CEA levels post-CRT was a useful predictor of pCR in LARC 21. In our current study, a post-CRT CEA level ≤2 ng/ml was significantly associated with a higher pCR rate (28.5% *vs*. 20.3%, *p*=0.037). Although the exact mechanism is unclear, we suppose that the lower post-CRT CEA level implies a lower tumor burden and, subsequently, less residual tumor in the rectum after CRT.

Several retrospective studies have emphasized the role of a longer interval between CRT and surgery in achieving a histological tumor response 22,23. Our study showed that an interval of >7 weeks between completion of CRT and surgical resection significantly increased pCR rate (26.9% *vs*. 18.3%, *p*=0.02). A previous study also reported that an interval >7 weeks could improve pCR after assessing 132 patients with LARC (35% *vs*. 17%, *p*=0.03) 24. Recently, a large retrospective study evaluating 17,255 patients from the National Cancer Data Base in America indicated that an interval greater than 8 weeks was associated with higher odds of pCR (OR=1.12, 95% CI=1.01-1.25, *p*=0.040) 25. However, the optimal timing for surgery after neoadjuvant CRT in LARC remains controversial. Stein et al. suggested that there was no incremental benefit from delaying surgery after CRT for tumor regression 26. To settle this issue, a prospective randomized trial named GRECCAR-6 was designed and the final result aims to identify the optimal interval for delivering pCR 27.

It is well known that TNM system category is closely associated with the prognosis of colorectal cancer. Some previous studies have found that pretreatment clinical N stage is an independent clinical predictor for achieving pCR 12,28. To date, few studies have assessed whether post-CRT TNM stage could be used to predict tumor response to CRT 13,29. To identify useful predictors for pCR, a recent study screened yc T stage (*p*<0.001) and yc N stage (*p*<0.001) as predictors of pCR after CRT 30. After assessing post-CRT clinical parameters, we found similar results, namely, that ycT stage 0-2 (OR=2.098, 95% CI=1.023-4.304, *p*=0.043) and ycN stage 0 (OR=2.011, 95% CI=1.264-3.201, *p*=0.003) are both independent indicators for pCR. To the best of our knowledge, lower clinical T and N stages are associated with less tumor burden in primary organs and lymph nodes. Further studies should be conducted to confirm the authentic values of ycT stage and ycN stage as indicators of pCR.

We would like to acknowledge several limitations in the present study. The first limitation is the retrospective methodology from a single-institution experience. Additionally, some post-CRT variables, including tumor gross change, circumferential extent of tumor and clinical stage shift after CRT, could not be fully evaluated. Furthermore, the patients included in our study were treated over a course of 11 years. This is a long time and CRT regimens and methods of pathologic assessment have changed in this period. However, even when considering the above limitations, our study identified several post-CRT indicators for pCR. External validation using another large database or prospective clinical studies would be of value. Based on our results, individualized therapy can be tailored for patients after CRT. For instance, patients with lower rectal cancer who are confirmed as complete responders to preoperative CRT may choose a wait-and-see policy with avoidance of abdominal perineal resection, as such patients may not achieve sufficient benefit from surgery. On the contrary, patients who fail to achieve a good response tend to have a poor prognosis and consequently need more intensive treatments or the application of novel therapies to minimize the possibility for local and distant recurrences.

This large, retrospective study identified post-CRT clinical parameters of considerable value as indicators for pCR. Post-CRT clinical (yc) T stage 0-2, ycN stage 0, an interval from the completion of CRT to surgery of >7 weeks and post-CRT CEA ≤2 ng/ml could be useful for predicting pCR. These indicators may help clinicians evaluate the efficiency of preoperative CRT and formulate individualized treatment strategies before surgery.

## AUTHOR CONTRIBUTIONS

Peng J, J Lin and QiuM designed the study and wrote the first draft. Wu X, Lu Z, Chen G, Li L, Ding P, Gao Y, Zeng Z and Zhang H performed the statistical analyses and interpretation. Pan Z and Wan D participated in study coordination and drafted the study protocol. All authors read and approved the final manuscript.

## Figures and Tables

**Table 1:** Demographics of patients who underwent preoperative chemoradiotherapy followed by surgery and their tumor characteristics (n=544).

Variable	Total (n=544,%)	pCR (n=126,%)	Non-pCR (n=418,%)	*p*-value
Gender				0.895
Male	361 (66.4)	83 (65.9)	278 (66.5)	
Female	183 (33.6)	43 (34.1)	140 (33.5)	
Age (years)^a^	55 (12-81)	55 (30-75)	55 (12-81)	0.546
Pre-CRT BMI (kg/m^2^)^b^	22.3±3.1	22.4±2.8	22.3±3.2	0.861
Pre-CRT DAV (cm)^a^	5 (0-15)	5 (1-12)	5 (0-15)	0.442
Tumor differentiation				0.001
Well	50 (9.2)	30 (23.8)	20 (4.8)	
Moderate	370 (68.0)	70 (55.6)	300 (71.8)	
Poor	124 (22.8)	26 (20.6)	98 (23.4)	
cTNM stage				0.147
II	170 (31.2)	46 (36.5)	124 (29.7)	
III	374 (68.8)	80 (63.5)	294 (70.3)	
Chemotherapy regimen				0.228
XELOX	473 (87.0)	120 (95.2)	353 (84.4)	
FOLFOX	49 (9.0)	4 (3.2)	45 (10.8)	
Capecitabine	22 (4.0)	2 (1.6)	20 (4.8)	
Radiation dose (Gy)	50 (30-70)	50 (30-50)	46 (30-70)	0.041
Radiation pattern				0.198
3D-CRT	230 (42.3)	47 (37.3)	183 (43.8)	
IMRT	314 (57.7)	79 (62.7)	235 (56.2)	
Post-CRT BMI (kg/m^2^)^b^	22.1±3.4	22.1±2.9	22.1±3.4	0.937
ycT stage				0.002
0	6 (1.1)	6 (4.8)	0	
1	7 (1.3)	2 (1.6)	5 (1.2)	
2	36 (6.6)	10 (7.9)	26 (6.2)	
3	334 (61.4)	81 (64.3)	253 (60.5)	
4	161 (29.6)	27 (21.4)	134 (32.0)	
ycN stage				0.002
0	323 (59.4)	89 (70.6)	234 (56.0)	
1	209 (38.4)	35 (27.8)	174 (41.6)	
2	12 (2.2)	2 (1.6)	10 (2.4)	
ycTNM stage				0.001
0	6 (1.1)	6 (4.8)	0	
I	35 (6.4)	8 (6.3)	27 (6.5)	
II	276 (50.7)	74 (58.7)	202 (48.3)	
III	214 (39.3)	37 (29.4)	177 (42.3)	
IV	13 (2.4)	1 (0.8)	12 (2.9)	
Post-CRT DAV (cm)^a^	5 (0-15)	5 (1-12)	5 (0-15)	0.157
Post-CRT CEA (ng/ml)^a^	2.4 (0.38-387.9)	2.1 (0.4-11.7)	2.5 (0.38-387.9)	0.011
Interval from completion of CRT to surgery (weeks)^a^	7 (1-12)	8 (5-12)	7 (1-12)	0.007
Surgical procedure				0.506
Anterior resection	318 (58.5)	81 (64.3)	237 (56.7)	
Abdominal perineal resection	196 (36.0)	44 (34.9)	152 (36.4)	
Hartmann procedure	14 (2.6)	1 (0.8)	13 (3.1)	
Palliative colostomy	16 (2.9)	0	16 (3.8)	

BMI: body mass index, DAV: distance of inferior tumor margin from the anal verge, CEA: carcinoembryonic antigen, pCR: pathologic complete response, cTNM stage: clinical tumor-node-metastasis classification, ycT stage: clinical tumor stage after chemoradiotherapy, ycN stage: clinical node stage after chemoradiotherapy, ycTNM stage: clinical tumor-node-metastasis classification after chemoradiotherapy, 3D-CRT: three-dimensional conformal radiation therapy, IMRT: intensity-modulated radiation therapy, CRT: chemoradiotherapy

a These values are presented as the median followed by the range in parentheses.

b These values are presented as the mean followed by the standard deviation in parentheses.

**Table 2:** Univariate analyses of indicators for pathological complete response to preoperative chemoradiotherapy in locally advanced rectal cancer.

Variable	pCR n=126 (% of total)	Non-pCR n=418 (% of total)	Odds ratio (95% CI)	*p*-value
Gender				0.895
Male	83 (23.0)	278 (77.0)	Reference	
Female	43 (23.5)	140 (76.5)	1.029 (0.676-1.567)	
Age (years)				0.546
≤60	83 (24.0)	263 (76.0)	Reference	
>60	43 (21.7)	155 (78.3)	0.879 (0.579-1.336)	
Post-CRT BMI (kg/m^2^)^a^				0.143
≤25	103 (35.4)	188 (64.6)	Reference	
>25	23 (22.1)	81 (77.9)	0.655 (0.372-1.53)	
Chemotherapy regimen				0.129
Capecitabine	2 (0.09)	20 (0.91)	Reference	
XELOX and FOLFOX	124 (23.8)	398 (76.2)	3.116 (0.718-13.516)	
Radiation pattern				0.198
3D-CRT	47 (20.4)	183 (79.6)	Reference	
IMRT	79 (25.2)	235 (74.8)	1.309 (0.869-1.971)	
Dose of radiotherapy (Gy)				0.044
<50	51 (19.4)	212 (80.6)	Reference	
≥50	75 (26.7)	206 (73.3)	1.513 (1.010-2.267)	
ycT stage				0.006
3-4	108 (21.8)	387 (78.2)	Reference	
0-2	18 (36.7)	31 (63.3)	2.414 (1.281-4.547)	
ycN stage				0.002
1-2	37 (16.7)	184 (83.3)	Reference	
0	89 (27.6)	234 (72.4)	1.952 (1.267-3.009)	
Post-CRT DAV (cm)				0.044
>5	73 (23.5)	283 (76.5)	Reference	
≤5	53 (28.2)	135 (71.8)	1.522 (1.011-2.291)	
Tumor differentiation				0.510
Well and moderate	100 (23.8)	320 (76.2)	Reference	
Poor	26 (21.0)	98 (79.0)	0.849 (0.522-1.382)	
Interval from completion of CRT to surgery (weeks)				0.020
≤7	43 (18.3)	192 (81.7)	Reference	
>7	83 (26.9)	226 (73.1)	1.640 (1.082-2.485)	
Post-CRT CEA (ng/ml)^b^				0.037
>2	64 (20.3)	251 (79.7)	Reference	
≤2	53 (28.5)	133 (71.5)	1.563 (1.027-2.379)	

CI: confidence interval, BMI: body mass index, DAV: distance of inferior tumor margin from the anal verge, CEA: carcinoembryonic antigen, pCR: pathologic complete response, ycT stage: clinical tumor stage after chemoradiotherapy, ycN stage: clinical node stage after chemoradiotherapy, 3D-CRT: three-dimensional conformal radiation therapy, IMRT: intensity-modulated radiation therapy, CRT: chemoradiotherapy

a There were only 395 patients with complete data for post-CRT BMI.

b There were only 501 patients with complete data for post-CRT CEA.

**Table 3:** Multivariate analyses of indicators for pathological complete response to preoperative chemoradiotherapy in locally advanced rectal cancer.

Variable	*p*-value	Odds ratio	95% CI of odds ratio
ycT stage 0-2	0.043	2.098	1.023-4.304
ycN stage 0	0.003	2.011	1.264-3.201
Interval from completion of CRT to surgery >7 weeks	0.010	1.795	1.151-2.801
Post-CRT CEA ≤2 ng/ml	0.038	1.579	1.026-2.432
Post-CRT DAV ≤5 cm	0.068	1.508	0.970-2.345
Radiation dose ≥50 Gy	0.086	1.515	0.942-2.436

CI: confidence interval, DAV: distance of inferior tumor margin from the anal verge, CEA: carcinoembryonic antigen, ycT stage: clinical tumor stage after chemoradiotherapy, ycN stage: clinical node stage after chemoradiotherapy, CRT: chemoradiotherapy
